# Cost of emergency hospital admissions to acute general wards for mental health problems among children and young people in England, 2012–2022: a retrospective observational study

**DOI:** 10.1136/bmjopen-2025-107143

**Published:** 2026-05-19

**Authors:** Hanifa Pilvar, Francesca Cornaglia, Joseph L. Ward, Adriana Vazquez-Vazquez, Kirsty Phillips, Kate Settle, Faith Gibson, Dasha Nicholls, Damian Roland, Helen Roberts, Russell M. Viner, Lee D. Hudson

**Affiliations:** 1 Nuffield Department of Primary Care Health Sciences, University of Oxford, Oxford, UK; 2 School of Economics and Finance, Queen Mary University of London, London, UK; 3 Population, Policy and Practice, UCL Great Ormond Street Institute of Child Health, London, UK; 4 Department of Women and Children’s Health, King’s College London, London, UK; 5 Great Ormond Street Hospital for Children, London, UK; 6 School of Health Sciences, University of Surrey, Guildford, UK; 7 Department of Brain Sciences, Imperial College London, London, UK; 8 SAPPHIRE Group, Population Health Sciences, University of Leicester, Leicester, UK; 9 Paediatric Emergency Medicine Leicester Academic (PEMLA) Group, Children’s Emergency Department, Leicester Royal Infirmary, Leicester, UK; 10 Great Ormond Street Hospital, London, UK

**Keywords:** Child, MENTAL HEALTH, PAEDIATRICS, Health Care Costs, Adolescent

## Abstract

**Abstract:**

**Objectives:**

To examine trends in the frequency and costs of emergency hospital admissions in acute wards for mental health conditions among children and young people in England between 2012 and 2022 and to assess socioeconomic and geographic disparities in these costs.

**Design:**

Retrospective observational cohort study using routinely collected administrative data.

**Setting:**

Secondary care acute wards; analysis includes all National Health Service (NHS) hospital admissions in England.

**Participants:**

All emergency hospital admissions in acute wards for individuals aged 5–18 years with a primary or secondary mental health diagnosis recorded between 2012 and 2022. Exclusion criteria included admissions without a mental health diagnosis or outside the defined age range.

**Primary and secondary outcome measures:**

Primary outcomes were the annual number and total cost of mental health-related emergency admissions. Secondary outcomes included length of stay, diagnostic categories contributing to cost, and variation by socioeconomic deprivation and geographic location.

**Results:**

Between 2012 and 2022, the total cost of emergency admissions for mental health among children and young people rose markedly, driven by increases in both admission rates and length of stay. Children from the most socioeconomically deprived areas experienced higher admission rates and greater associated costs. Substantial regional variation in the financial burden was also observed. Eating disorders and self-harm were the main diagnostic categories contributing to the rise in costs. Following the COVID-19 pandemic, total admission numbers declined, but overall costs remained high due to a shift in diagnostic mix towards conditions associated with longer hospital stays and higher per-admission costs.

**Conclusions:**

The increasing financial burden of paediatric mental health crises highlights the urgency of addressing upstream drivers of poor mental health. Policies should prioritise early intervention, reduce regional and socioeconomic disparities, and ensure equitable allocation of mental health resources. Further research should explore the effectiveness of community-based alternatives to hospital care.

**STRENGTHS AND LIMITATIONS OF THIS STUDY**

- This study uses national-level Hospital Episode Statistics (HES) data covering all emergency admissions for children and young people in England over a 10-year period, providing a comprehensive and nationally representative dataset.
- The inclusion of detailed demographic, geographic and hospital stay variables enables stratified analyses and exploration of variation across population subgroups.
- A key limitation is that HES data are collected for administrative purposes, which may result in under-reporting of secondary diagnoses and variation in data quality across providers.
- Cost estimates are derived from the National Cost Collection (NCC), which is subject to variability in reporting practices across National Health Service (NHS) trusts and excludes certain one-off or indirect costs.
- The use of national average unit costs and older healthcare resource group (HRG4) codes may reduce the precision of cost estimates and mask local variation in service use and efficiency

## Introduction

 There is growing concern regarding the global prevalence of mental health disorders among children and young people (CYP)^1^. In England, one in six CYP is now experiencing substantial psychological distress, an increase from one in nine in 2017.^2^ This trend is reflected in the rising rates of self-harm, eating disorders and acute mental health crises, all of which contribute to the economic argument for investing in mental health prevention and interventions.^3^

Against this background, recent evidence has shown large increases in the number of CYP with mental health concerns presenting to emergency departments and requiring admission to acute medical wards.^4 5^ We recently reported that in England, between 2012 and 2022, there was a 65% increase in all emergency admissions due to mental health concerns, and a 518% increase in those due to eating disorders. These increases are likely to be due to a range of reasons such as higher background prevalence of mental health disorders, higher level of severity at presentation, long waits for specialist mental health beds, and limited capacity in community mental health settings to support CYP in crisis outside of a hospital setting.^4^,^6^ Regional disparities in costs and sociodemographic variations further underscore the complexities of mental health service provision across England.^7^ An increased reliance on acute medical wards for mental health-related admissions inevitably places substantial strain on hospital inpatient capacity, and wards may lack the infrastructure and trained staff needed to provide appropriate care to these CYP. This leads to unmet needs and increased hospitalisations for CYP.^8^

The economic burden of mental health conditions on national healthcare systems is well documented. In the UK, the total economic impact of mental illness has been estimated at £118 billion per year, incorporating direct National Health Services (NHS) costs, lost productivity and social care expenses. Some 6% of this cost is attributed to children under 14 years of age.^9^ Moreover, recent research indicates that mental health difficulties in adolescence have the potential to have long-term financial consequences, increasing healthcare costs well into adulthood.^10^ Despite this, little is known about the immediate economic burden of emergency mental health admissions for CYP within the NHS.

Most cost-effectiveness studies in child and adolescent mental health have focused on specific interventions or preventive measures,^11^ such as school-based screening programmes,^12^ or broader cost analyses in adult populations.^13^ To our knowledge, no study has systematically measured the direct costs of emergency mental health admissions for CYP in acute medical settings. Despite their importance, the economic implications of these admissions remain poorly understood. These high costs are a crucial part of the overall impact of such admissions and may limit the ability to redirect funding towards developing and delivering alternative models of care.

We examined the financial costs associated with emergency admissions to hospital due to mental health concerns among CYP in England between the financial years 2012/2013 and 2021/2022. For the first time, we systematically break down these costs, providing crucial insights for policymakers. We used linked data from the Hospital Episode Statistics (HES) and National Cost Collection (NCC) databases to analyse variations in costs across demographic groups, diagnoses and geographic regions.

## Methodology

### Data

We used HES data, which captures 97% of hospital activity in England,^5^ to identify CYP admitted to an acute medical ward due to a mental health concern. Data were available for all emergency hospital admissions among CYP aged 5 to 18 years from 1 April 2012 to 31 March 2022.

We used the NCC database to estimate the cost of admissions due to mental health concerns identified within HES. These data provide national average unit costs for NHS services derived from cost data submitted by NHS trusts. These figures reflect the average cost of delivering care from the perspective of healthcare providers and do not represent reimbursement tariffs, actual payments, insurance claims or patient-level charges. As the NHS is a tax-funded system, patients do not incur direct charges for care, and costs therefore represent resource use within the healthcare system rather than individual expenditure.

As outlined in the Health and Social Care Act of 2012,^14^ NHS services are priced using a system based on “currencies”—units of payment specific to healthcare services. For admitted patient care and accident and emergency (A&E) services, the relevant currency is the healthcare resource group (HRG), which categorises diagnoses and interventions requiring similar resource levels. With around 26 000 individual codes to represent specific diagnoses and treatments, these are grouped into HRGs to establish currencies that are practical in calculating costs. The most recent version, HRG4+, consists of more than 2800 currencies (as of 2021/2022). Each HRG represents a complete “spell of care”, from admission to discharge.

Using Secondary Uses Service (SUS) HRG codes available in the HES dataset, we matched these to the corresponding currency codes in the NCC data to derive the cost of each admission identified as being due to a mental health concern. This process utilised national average unit costs from the NCC database for the period 2012/2013 to 2021/2022, enabling us to calculate the financial burden associated with mental health admissions.

### Analysis

We first identified emergency admissions due to mental health concerns using the primary reason for admission in HES, coded to the International Classification of Diseases 10th Revision (ICD-10), as in our previous study.^5^ We categorised these admissions using a modification of the Global Burden of Disease (GBD) 2021 cause hierarchy^15^ as those due to “anxiety”, “depression”, “eating disorders”, “substance misuse” and “other mental health disorders”. Self-harm is not recorded as a primary reason for admission in HES, but as one of up to 20 additional causes, with the primary cause coded as an injury resulting from self-harm. We coded admissions as “self-harm” where the primary reason for admission was an injury or external cause (ICD-10 chapters T, S, V, X, Y), and where self-harm was recorded as the first additional reason for admission. This operational definition reflects English administrative coding practices and may limit direct comparability with studies from other countries that use different coding conventions. We then classified all remaining admissions where the treating clinician was from a mental health specialty, or where the primary reason for admission was coded within chapter 5 of ICD-10 (mental and behavioural disorders), within the “other mental health disorders” category.

We calculated the number of admissions by length of stay. Length of stay was classified as less than 2 days (short stay) or 2 days or longer (long stay) to align with the definition of length of stay used in the NCC data.^16^ Short stays, typically involving fewer than 24 hours of hospitalisation, are often associated with less severe conditions, same-day procedures or emergency admissions that do not require extended inpatient care. These admissions generally involve minimal treatment and nursing care compared with longer stays. We merged the number of admissions with non-elective inpatient reference cost data by year, currency code and length of stay (short vs long). On average, 10% of unit costs were missing after the merge, most of which were from the period 2014/2015–2016/2017, when the calculation of unit costs was transitioning from a top-down to a bottom-up approach in most NHS trusts. In 2013, the NHS began encouraging trusts to adopt patient-level information and costing systems (PLICS) to facilitate the bottom-up costing approach rather than the traditional top-down approach based on average costs.^17^

To address this, we substituted missing unit costs with the most recent available unit cost (since 2009) for the corresponding currency code and length of stay. After this adjustment, the final number of admissions with missing unit costs accounted for less than 0.1% of total admissions. For further details on the number and proportion of mental health admissions with missing unit costs for each financial year before and after substitution see online supplemental table A1. We then used national average unit cost by year, currency code and length of stay multiplied by total number of admissions to calculate the cost. We adjusted all costs for inflation using the Consumer Prices Index including owner occupiers’ housing costs (CPIH), expressed in constant 2015 prices.

We report cost of admissions by mental health concern, and by sex, age, ethnicity, cause of mental health concern, Index of Multiple Deprivation (IMD) and Integrated Care Board (ICB), which are NHS organisations responsible for planning healthcare services for local populations.^18^ ICBs are statutory NHS organisations established in England in July 2022 under the Health and Care Act 2022, replacing clinical commissioning groups and taking on local responsibility for planning and arranging NHS services within defined geographic areas. ICBs bring together NHS trusts, general practices, local authorities and other partners to set strategic priorities, plan health services, allocate resources and oversee performance for the populations they serve. We have defined the geographic boundaries of ICBs and have used them for the entire period of the study to show the geographic variation in the cost.

We used sex recorded within HES and categorised individuals as female or male. In England, almost all CYP aged under 16 years are admitted to paediatric wards, but criteria for older CYP are highly variable, with CYP aged 16–18 years often admitted to adult wards. We characterised age as 5– 10, 11–15 and 16–18 years (note that in England almost all CYP aged under 16 years will be admitted to paediatric wards, but criteria for admitting 16–18-year-olds is highly variable, with many admitted to adult wards).^19^ Ethnicity was classified as white, black, South Asian, mixed, other or not specified. Deprivation was assessed using the population-weighted IMD quintiles. Geographic variation within England was examined across the 42 NHS ICBs. Both IMD and ICB are defined based on the patient’s postal address.

### Ethics approval

Ethics approval was provided after review by London Brent NHS Research Ethics Committee on 14 November 2022 (reference 18/LO/1267).

### Patient and public involvement

This study is part of the broader Mental Health Admissions to Paediatrics Wards Study (MAPS), within which we undertook several engagement activities with CYP and families with lived experience of admission to general hospital wards for mental health concerns. These contributors were involved in informing the grant application, study design and interpretation of findings. In addition, individuals with lived experience are members of the project’s stakeholder and study advisory group. For this specific study, we also conducted focus groups with CYP to discuss the suitability and acceptability of using hospital administrative data to examine admissions for mental health concerns. Findings from this work have been and will continue to be disseminated through stakeholder networks, including patient and public contributors.

## Results

### Summary statistics

The characteristics of the population under study are presented in table 1. The population consists of 344 059 emergency admissions for CYP with mental health problems, with a majority of participants aged 11 to 15 years (52%) and 74% of the population being female. The ethnic composition is predominantly white (78%), with smaller shares from black (3%), South Asian (3%) and other ethnic groups. In terms of socioeconomic status, 24% of the sample falls in the most deprived IMD quintile, while 17% are in the least deprived. The most common diagnosis is self-harm (53%), followed by other diagnoses (32%), substance misuse (5%) and eating disorders (4%).

**Table 1:** Characteristics of the study population (mental health admissions of children and young people from 2012/2013 to 2021/2022)

Variable	Observations (total N=344 059) n (%)
Age (years)	
11–15	177 678 (52)
16–18	146 887 (43)
5–10	19 494 (5)
Sex	
Female	253 858 (74)
Male	90 201 (26)
Ethnicity	
Black	9049 (3)
White	269 888 (78)
Mixed	9191 (3)
South Asian	10 661 (3)
Other	11 704 (3)
Unknown/not specified	33 566 (10)
IMD quintile	
Quintile 1 (20% least deprived)	57 468 (17)
Quintile 2	72 305 (21)
Quintile 3	68 553 (20)
Quintile 4	63 644 (18)
Quintile 5 (20% most deprived)	82 089 (24)
Diagnosis	
Anxiety	12 253 (4)
Depression	7790 (2)
Eating disorder	13 449 (4)
Self-harm	181 165 (53)
Substance misuse	20 497 (5)
Other	108 905 (32)

Notes: The table shows the number of observations of children and young people (CYP) admitted for emergency mental health problems in general paediatric wards from 2012/2013 to 2021/2022, broken down by demographic, socioeconomic and diagnosis characteristics. Deprivation was measured using the Index of Multiple Deprivation (IMD), an area-level composite measure of relative deprivation in England. IMD is based on seven weighted domains: income, employment, education, health, crime, barriers to housing and services, and living environment. Patients were assigned to national IMD quintiles based on their area of residence, with Quintile 1 representing the least deprived 20% of areas and Quintile 5 the most deprived 20%.

IMD, Index of Multiple Deprivation.

### Cost analysis

**Figure 1:**
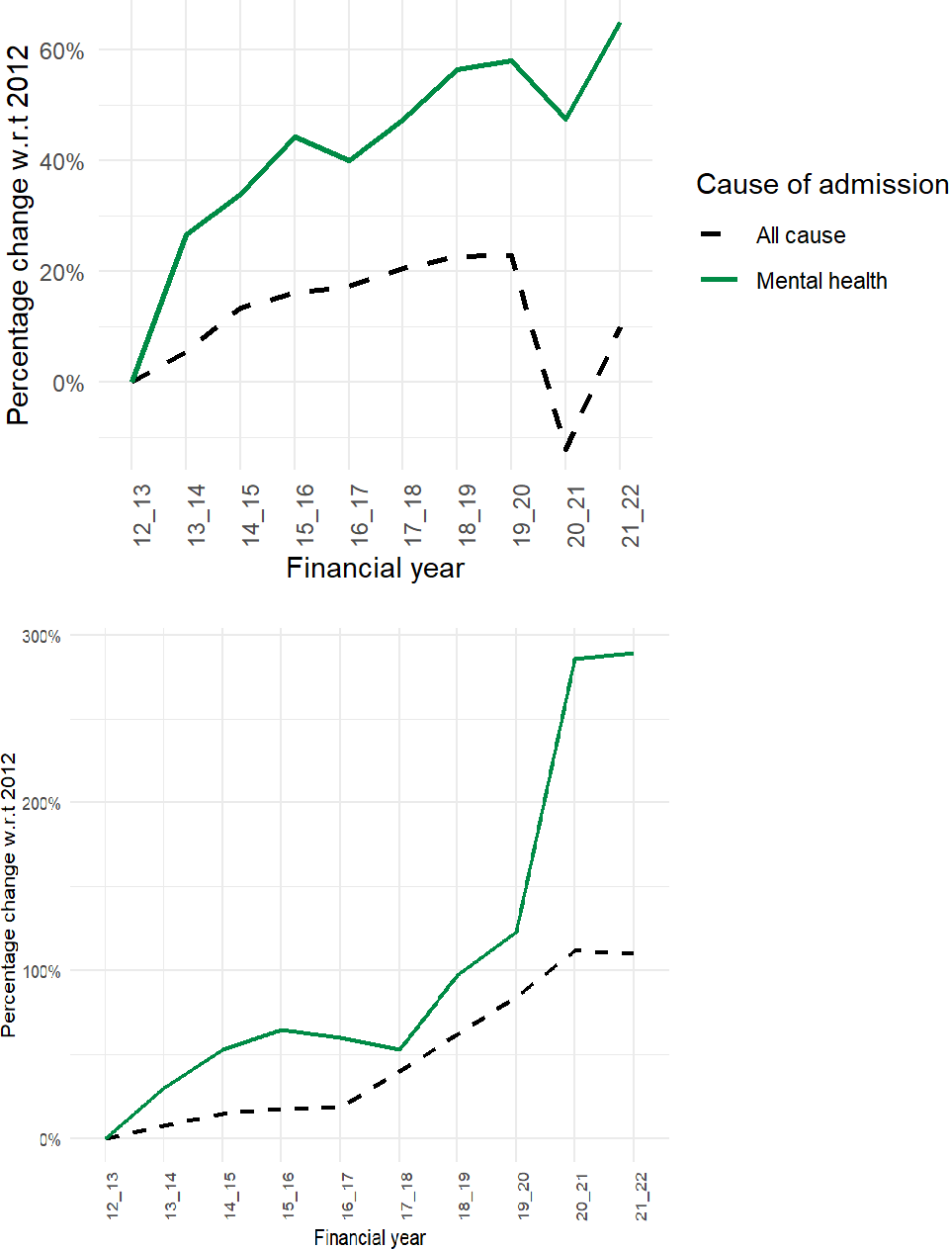
Year-on-year percentage change in the number of admissions (top panel) and the cost (bottom panel) of all-cause emergency paediatric and emergency mental health admissions among children and young people (all costs are expressed in constant 2015 prices).

In this section, we present the time trends in the cost of CYP emergency mental health admissions to general wards in England, comparing them with total paediatric emergency admissions where relevant. Mental health admissions among CYP have been increasing at a faster rate than all-cause paediatric emergency admissions. While all-cause paediatric emergency admissions rose by 10%, from 311 058 in 2012/2013 to 342 511 in 2021/2022, mental health emergency admissions for CYP increased by 65%, growing from 24 197 to 39 925 during the same period.^5^ The left panel of figure 1 illustrates the year-on-year changes and online supplemental figure A1 shows the overall levels of admissions.

The right panel of figure 1 shows that the cost of all-cause emergency admissions increased by 100% over the study period, while the cost of emergency mental health admissions rose much more rapidly, by just below 300%. The cost of paediatric emergency mental health admissions was £22.5 million in 2012/2013, which rose to £87.3 million in 2021/2022. All costs are adjusted for inflation using constant 2015 prices (see online supplemental figure A2). There was a noticeable spike in mental health admission costs after 2019/2020, followed by a flattening after 2020/2021, coinciding with the COVID-19 pandemic. Consequently, the share of mental health costs as a proportion of all-cause paediatric emergency admission costs grew from 7% in 2012/2013 to 13% by the end of the study period.

**Figure 2:**
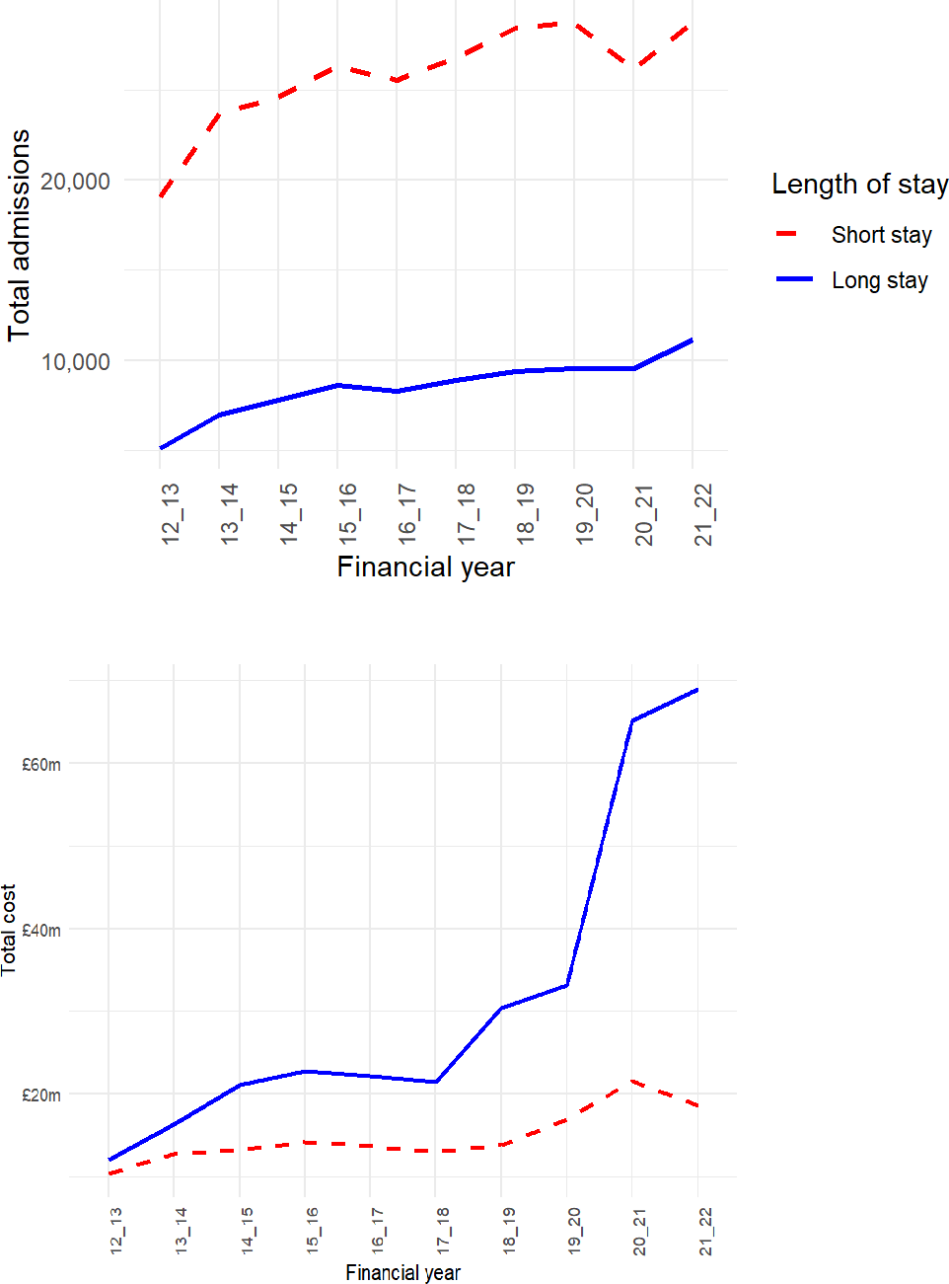
Number (top panel) and cost (bottom panel) of emergency mental health admisssions among children and young people by length of stay (all costs are expressed in constant 2015 prices.

To better understand the rising mental health costs of emergency admissions among CYP we examined admissions by length of stay in figure 2. Throughout the study period, the number of long-stay admissions ranged from one-quarter to one-half of short-stay admissions. However, long-stay admissions incurred substantially higher costs, particularly in recent years. By 2021/2022, the cost of long-stay admissions was four times higher than that of short-stay admissions. While the cost of short-stay admissions fluctuated, with declines in certain years, the cost of long-stay admissions consistently increased over the entire study period.

The plateau in costs observed after 2020/2021, despite a reduction in total admissions in figure 1, reflects a change in the composition of admissions. Specifically, following the COVID-19 pandemic, there was a decline in short-stay admissions and a concurrent increase in long-stay admissions, which are substantially more costly. This shift in case mix explains the observed divergence between trends in total admissions and overall costs shown in figure 1.

### Cost by patient characteristics

In this subsection, we examine how different groups contribute to the rising costs of mental health emergency admissions. Understanding variations across sex, age and ethnic groups helps to identify patterns in healthcare needs, access to services and underlying social factors. Additionally, analysing costs by diagnostic groups allows us to identify specific conditions driving the increase in mental health-related emergency admissions, providing valuable insights for resource allocation and service planning.

online supplemental figure A4 illustrates the trends in costs of mental health by sex, age, ethnicity and cause of mental health concern. Female CYP and those aged 11–15 years are the primary drivers of the cost increase. By 2021/2022, over 80% of the total mental health cost was attributed to females, and more than 50% to CYP aged 11–15 years (this corresponds to the share of this sex and age group of total mental health admissions; see online supplemental figure A3). Additionally, more than 70% of the total cost was associated with admissions of CYP from a white ethnic background, aligning with their share of the total number of admissions. This suggests that CYP from white ethnic backgrounds do not incur higher costs per admission compared with other ethnic groups. However, CYP with a white background have higher admission rates than their share in the study group, which might be due to better service access for this group.

In terms of diagnosis, eating disorders and self-harm were the two most costly conditions in 2021/2022, while substance misuse and depression accounted for the lowest costs. A comparison of costs with the number of admissions (see online supplemental figure A3) reveals that although eating disorders represent a relatively small share of total admissions, they are associated with substantially higher costs per admission. Additionally, there was a rapid rise in the cost of admissions for eating disorders after 2019/2020, contributing substantially to the overall increase in costs during the later years of the study period.

### Geographic variation of the cost

In this subsection, we examine geographic variation in the costs of mental health emergency admissions for CYP by analysing the index of IMD quintile and ICB of the patient’s residence

This analysis helps to explore potential disparities in healthcare costs across different socioeconomic groups and regions. Understanding these patterns can provide insights into how resources are distributed and used across areas, highlighting differences in both service demand and provision that can inform healthcare planning and policy.

**Figure 3:**
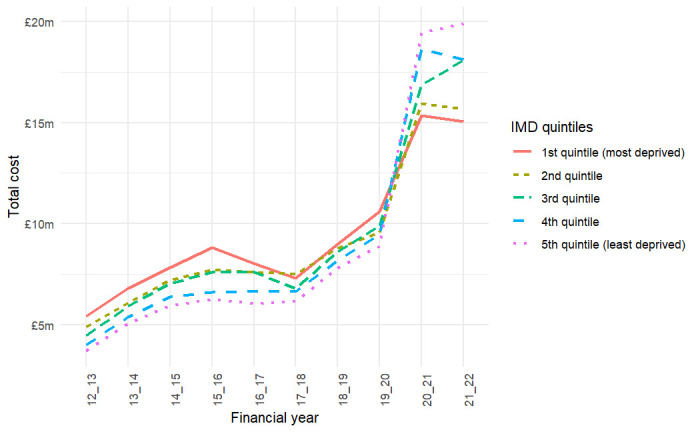
Cost of emergency mental health admissions among children and young people by Index of Multiple Deprivation (IMD) quintile of the residence of the patient (all costs are expressed in constant 2015 prices).

Figure 3 illustrates the trend in the costs by IMD quintile. CYP from the most deprived neighbourhoods consistently accounted for the highest number of admissions across all years (see online supplemental figure A5). However, the gap in admissions between the least and most deprived areas narrowed over time. For instance, in 2012/2013, admissions among CYP from the most deprived areas were 80% higher than those from the least deprived neighbourhoods. By 2021/2022, this gap had reduced to just 5%, indicating a rapid rise in admissions from the least deprived areas. This narrowing gap reflects a more rapid increase in admissions among CYP from the least deprived areas over time, rather than a decline in admissions from the most deprived neighbourhoods. This trend is descriptive and may be driven by multiple factors, including changes in help-seeking behaviour, thresholds for admission, and availability of community-based mental health services across socioeconomic groups.

In terms of the associated cost, before the COVID-19 pandemic, the majority of costs were attributed to admissions of CYP from the most deprived areas; however, this pattern changed after the pandemic. One factor contributing to the change in cost patterns after the pandemic is the declining share of long-stay admissions (the costliest) among the most deprived areas, whereas an opposite trend is evident in the least deprived neighbourhoods (see online supplemental figure A6).

online supplemental figure A7 presents the distribution of costs by ICB across England in the final year of our study. In 2012/2013, most ICBs had costs below £750 000, while by 2021/2022, the majority of ICBs incurred costs exceeding £1 million. Throughout the study period, ICBs in the North, South East and London consistently reported higher costs compared with other regions.

## Discussion

Our study provides a comprehensive analysis of the trends and costs associated with emergency mental health admissions among CYP in England between 2012 and 2022. Our findings indicate that alongside increases in overall mental health admissions compared with other causes, costs have increased nearly three times faster than all-cause paediatric emergency admissions. This highlights the growing financial strain on the NHS and the broader pressures on acute care services. Thus, our study confirms that increasing admissions translate into substantial cost pressures, with mental health-related emergency admissions accounting for an increasing share of total paediatric emergency costs. Our findings suggest that this financial strain is not solely driven by rising case numbers but also by prolonged hospital stays.

Long-stay admissions accounted for a disproportionate share of costs, a finding consistent with previous evidence on the impact of prolonged inpatient care in mental health settings.^7^ Notably, admissions for eating disorders and self-harm (the most common) contribute most substantially to cost increases, despite eating disorders representing a relatively small proportion of overall admissions. This suggests that conditions requiring intensive and prolonged care are major cost drivers within CYP mental health emergency admissions, particularly among females, where eating disorders are much more common^20^.

Following the pandemic, while the number of admissions declined, the associated costs did not decrease accordingly and instead remained relatively flat. One possible explanation for this divergence is a shift in the composition of cases. As shown in online supplemental figure A3, panel d (online supplemental appendix), the overall drop in admissions appears to be primarily driven by a reduction in ‘Other’ diagnoses. In contrast, admissions for eating disorders and self-harm—conditions typically associated with higher costs—did not decline and, in fact, increased after the pandemic.

Our findings add to the clinical contexts of acute hospital settings increasingly absorbing the burden of CYP mental health crises, despite being poorly equipped to manage them.^4^ Evidence indicates that acute hospital services are increasingly managing CYP presenting in mental health crisis, despite being primarily designed for physical healthcare rather than specialist mental health treatment. In England, referrals to child and adolescent mental health services (CAMHS) more than doubled between 2017 and 2022, signalling a substantial increase in underlying need. While investment in CYP’s mental health services has risen over this period, it has not matched the growth in demand. Consequently, a growing share of referrals has been closed before treatment, reversing previous improvements, and waiting times for those accepted into care have increased. These pressures on community and specialist services may be contributing to more CYP presenting in crisis to emergency departments and being admitted to acute medical wards, which may partly account for the rising admissions and costs observed in this study.^5^ Associated high costs are an important part of the argument for strengthening and redeveloping pathways of care as a priority. This could include greater investment in community-based services, early intervention programmes and crisis care alternatives. Evidence on alternatives to admission to acute hospital settings for CYP experiencing a mental health crisis remains limited^6 21^ and heterogeneous. However, a systematic review by Clisu *et al* identified some evidence that in-home interventions, particularly multisystemic therapy, may be associated with improved psychological outcomes and shorter lengths of hospital stay, although many CYP still required admission.^6^ Overall, the authors concluded that no single intervention could be recommended as a substitute for inpatient care, highlighting the need for closer alignment betweencommunity and inpatient services and for further high-quality research evaluating community-based crisis interventions. Furthermore, our findings add to the need for broader ways to address predisposers to mental health in CYP, in particular social determinants of health.^5^

Our study also highlights regional inequalities in the cost burden of CYP mental health emergency admissions. Admissions from more socioeconomically deprived areas were disproportionately associated with long hospital stays and higher per-admission costs, a pattern that mirrors broader inequalities in mental health outcomes.^10^

Targeted funding and resource allocation strategies could help reduce geographic disparities by directing investment toward regions where CYP face the highest unmet mental health needs. Policymakers should consider enhancing mental health support in schools, increasing early intervention resources, and addressing social determinants of health to reduce the need for costly emergency admissions.

Although our findings are based on data from England, which has a tax-funded NHS with no direct patient charges, they are likely to have relevance beyond the UK. Direct international comparisons are challenging because health systems differ substantially in psychiatric bed availability, the provision of community-based crisis services, and the role of insurance or reimbursement mechanisms in shaping admission thresholds and length of stay. Published data on admissions of CYP to acute medical wards for mental health crises in other countries are limited, and there is a paucity of longitudinal analyses outside the UK. However, a recent systematic review^4^ identified evidence of increasing mental health-related admissions among CYP across several geographic settings, suggesting that rising demand for acute care is not unique to England. Given the global increase in the incidence of child and adolescent mental health difficulties,^1 22^ it is plausible that acute care services in other high-income health systems are experiencing similar pressures, despite differences in service configuration. This underscores the importance of replicating similar analyses in other countries to better understand how health system design influences admission patterns, length of stay and costs.

The observed geographic variation in costs across ICBs should be interpreted as descriptive rather than causal. Our analysis does not directly measure supply-side factors such as the availability of child and adolescent psychiatrists, specialist mental health beds, community CAMHS capacity or crisis response services. As such, differences in costs between areas should be viewed as hypotheses regarding variation in service configuration, resource allocation and specialist workforce capacity, rather than as evidence of specific mechanisms. Future research linking hospital admission costs to local indicators of mental health service capacity, workforce availability and community crisis provision would substantially strengthen understanding of how system-level factors shape admission patterns, length of stay and costs, and would enhance the policy relevance of these findings.

### Study strengths and limitations

A key strength of our study is the use of national-level data (HES data) encompassing all hospital admissions for CYP in England over a 10-year period. This comprehensive dataset allows for robust analysis and ensures that the findings are nationally representative. The availability of detailed information on demographics, geographic regions and lengths of stay further strengthens the analysis and enables exploration of variation across different population groups and settings.

HES data have multiple limitations and are primarily collected for administrative and billing purposes rather than research. Conditions or diagnoses that are not the primary reason for admission may be under-reported. Some providers may fail to report accurate activities in certain periods due to technical issues.^23^ Additionally, specific groups, such as individuals with higher socioeconomic status who use private services or those who tend to avoid hospital care, may be under-represented in the dataset.

The NCC data have several limitations. One key issue is the variability in cost reporting across NHS trusts which could introduce inconsistencies, as similar healthcare services may have been assigned widely different costs. Furthermore, the exclusion of one-off expenditures and certain cost categories from the NCC dataset may result in an incomplete representation of healthcare costs, limiting the ability to fully capture the financial burden of services.^16^

Another limitation is the use of outdated HRG codes such as HRG4 for certain services in the HES data. We have converted HRG4+ codes in the NCC data to HRG4 where necessary to be compatible with HES data. HRG4+ has more granularity over HRG4 by introducing the scores of complexity and comorbidity (CC).^24^

Furthermore, our cost estimates rely on national average unit costs, which may obscure local variations in spending and service efficiency.^16^ Future research could explore trust-level variations in spending to better understand efficiency differences across the NHS.

Additionally, our study primarily focuses on direct hospital costs and does not account for long-term economic consequences, such as increased primary care utilisation following emergency admissions. Given the evidence that adolescent mental health issues have long-term financial impacts,^10^ future research should explore the broader economic costs of CYP mental health crises, including their effects on education, employment and social care systems.

Length of stay was categorised as less than 2 days or 2 days or more to align with NCC reporting. While this distinction captures broad differences in resource use, it conceals substantial heterogeneity within long-stay admissions, which may range from a few days to several weeks. As a result, policy interpretations of cost differences should recognise that long-stay admissions represent a diverse group of hospitalisations with potentially very different clinical pathways and resource requirements.

## Conclusions

The growing cost of emergency mental health admissions among CYP highlights an important perspective on the economic impacts of increased mental health admissions to general medical settings. Addressing more joined-up approaches to managing crisis outside the hospital where possible would provide a more financially sustainable approach. Addressing socioeconomic and geographic disparities in access to care is critical to ensuring equitable and efficient resource distribution across the NHS. Policymakers must consider targeted interventions and strategic investments to better support CYP with mental health needs while alleviating pressure on already strained acute care services. In the context of overall rising costs within the NHS and sustainability, we recommend addressing the particular pathways and costs of CYP with mental health needs, which represents a substantial increase in burden, as an important priority area for focus and development. Work on the time, social and emotional costs to CYP, their families and frontline practitioners is also needed.

## Supplementary material

10.1136/bmjopen-2025-107143 online supplemental file 1

10.1136/bmjopen-2025-107143 online supplemental file 2

10.1136/bmjopen-2025-107143 online supplemental file 3

10.1136/bmjopen-2025-107143 online supplemental file 4

10.1136/bmjopen-2025-107143 online supplemental file 5

10.1136/bmjopen-2025-107143 online supplemental file 6

10.1136/bmjopen-2025-107143 online supplemental file 7

10.1136/bmjopen-2025-107143 online supplemental file 8

## Data Availability

Data may be obtained from a third party and are not publicly available.
