# Supplementary figures and images for: Cost of emergency hospital admissions to acute general wards for mental health problems among children and young people in England, 2012–2022: a retrospective observational study
Source: BMJ Open. 2026 May 19;16(5):e107143. doi: 10.1136/bmjopen-2025-107143 (PMC13202060; doi:10.1136/bmjopen-2025-107143)

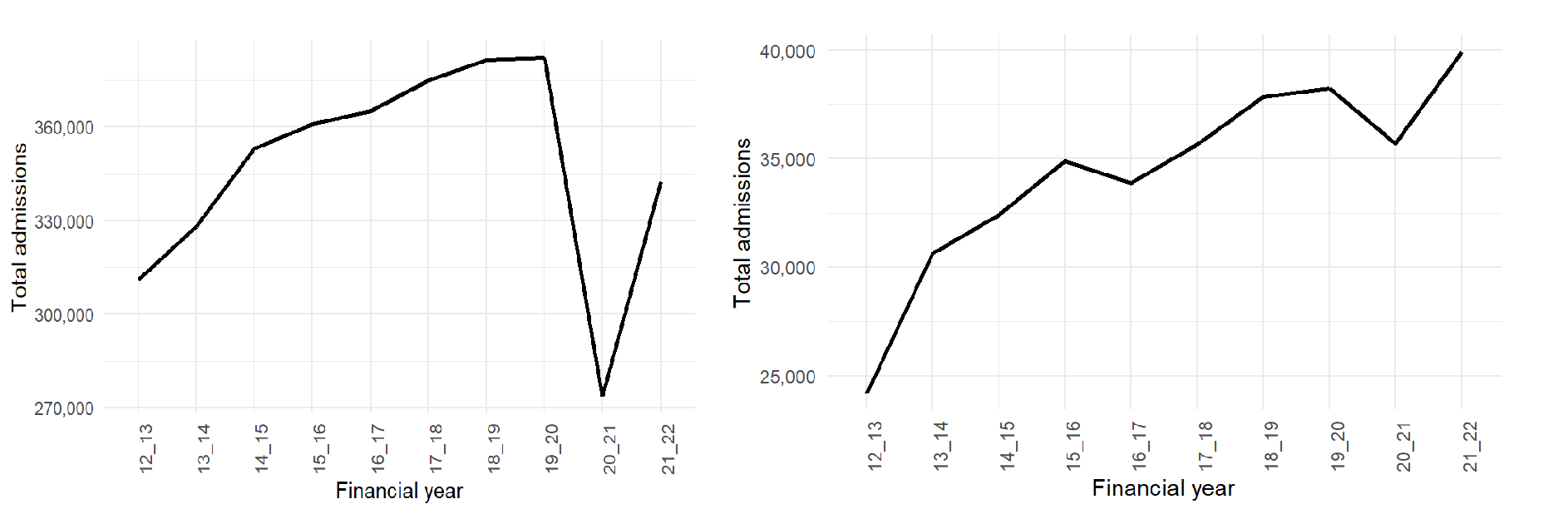

Supplement: online supplemental file 1 [file bmjopen-16-5-s001.png]

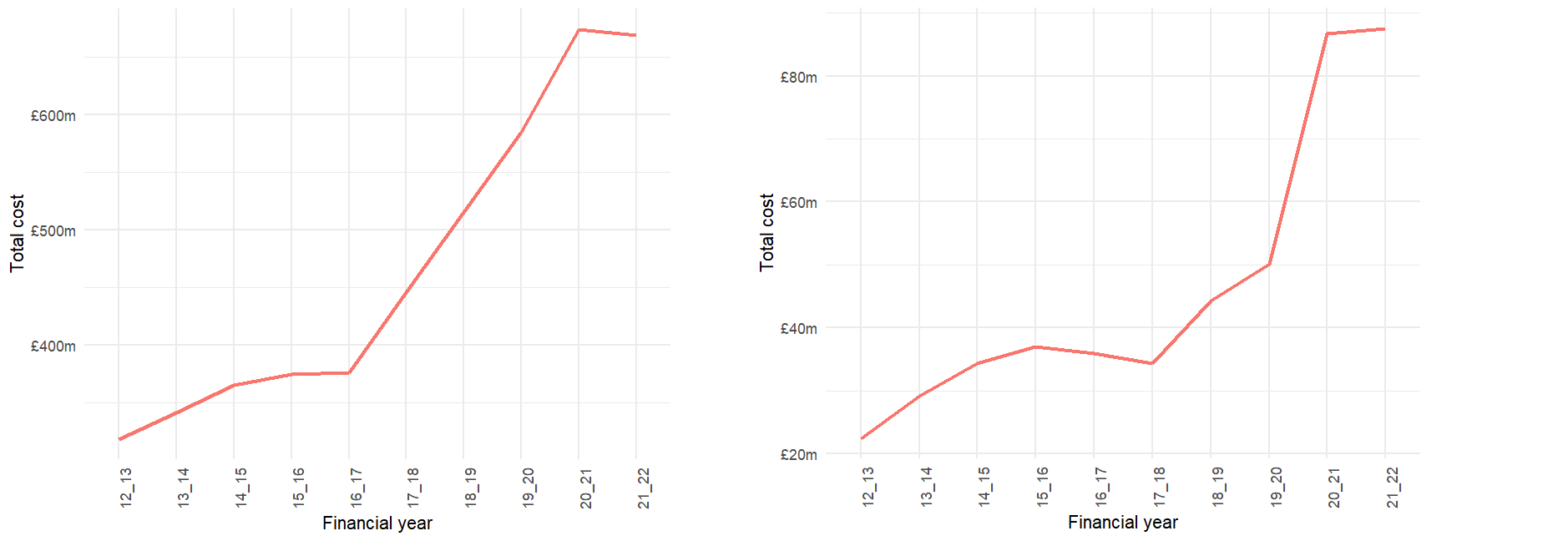

Supplement: online supplemental file 2 [file bmjopen-16-5-s002.png]

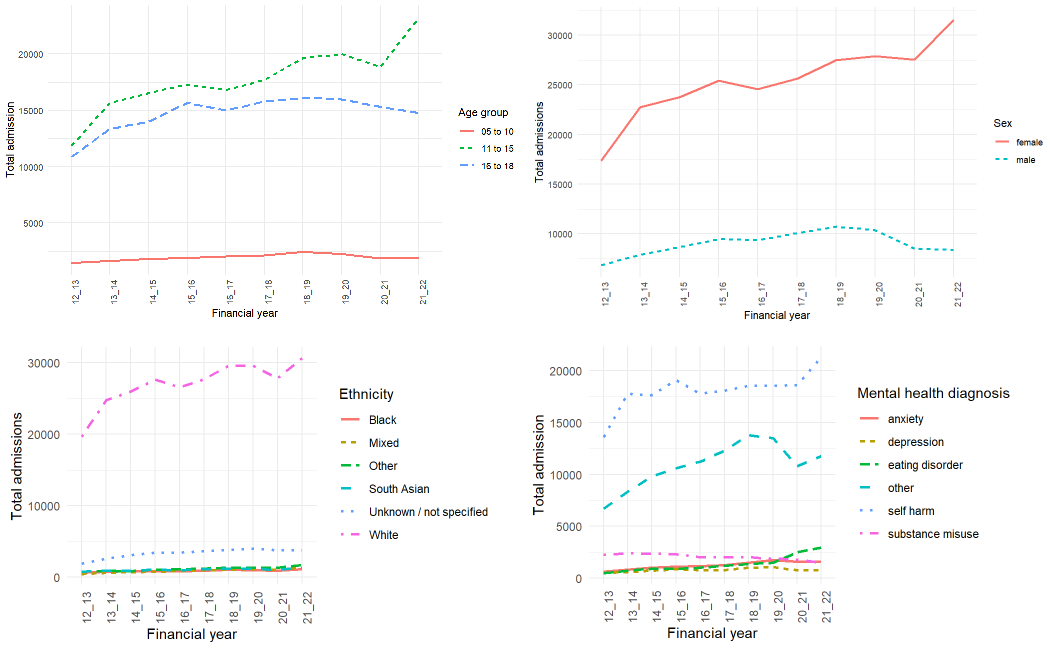

Supplement: online supplemental file 3 [file bmjopen-16-5-s003.png]

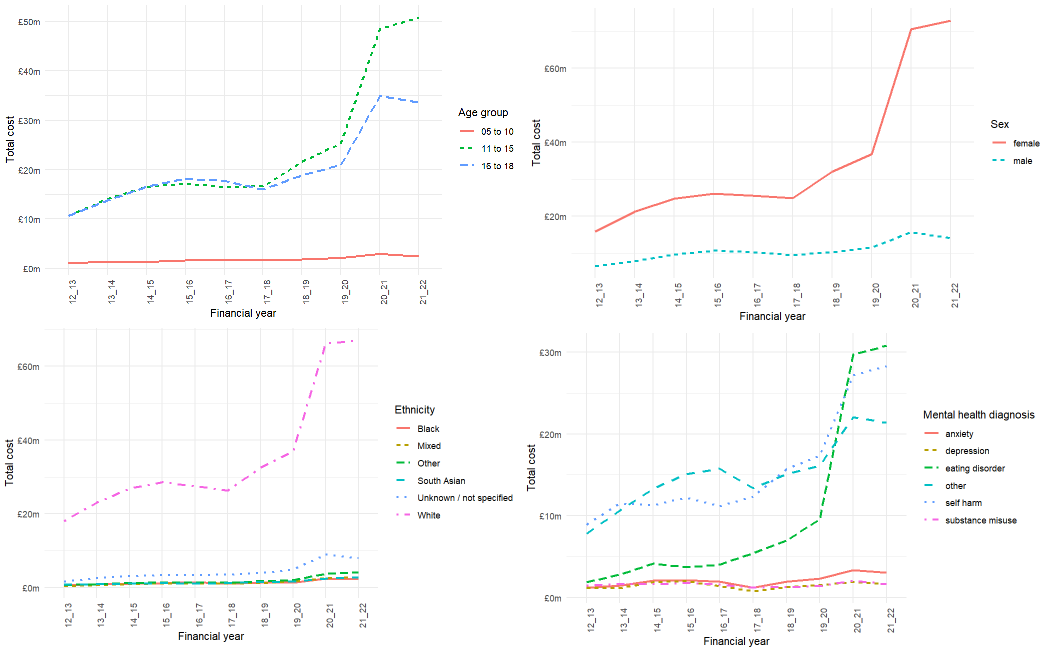

Supplement: online supplemental file 4 [file bmjopen-16-5-s004.png]

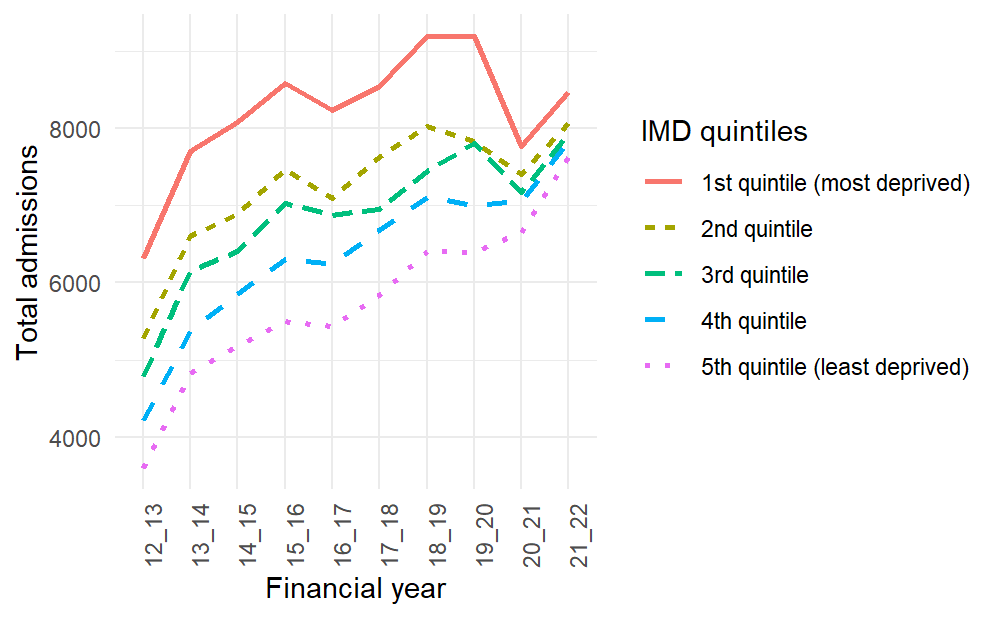

Supplement: online supplemental file 5 [file bmjopen-16-5-s005.png]

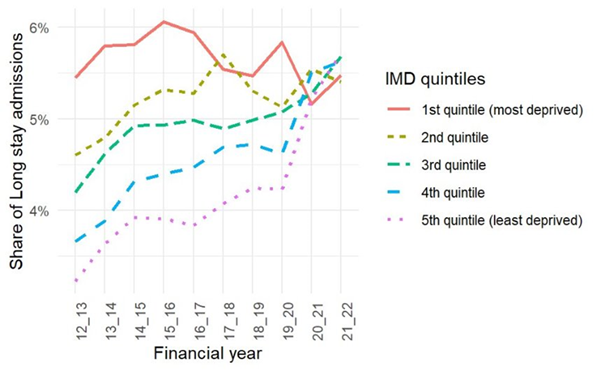

Supplement: online supplemental file 6 [file bmjopen-16-5-s006.png]

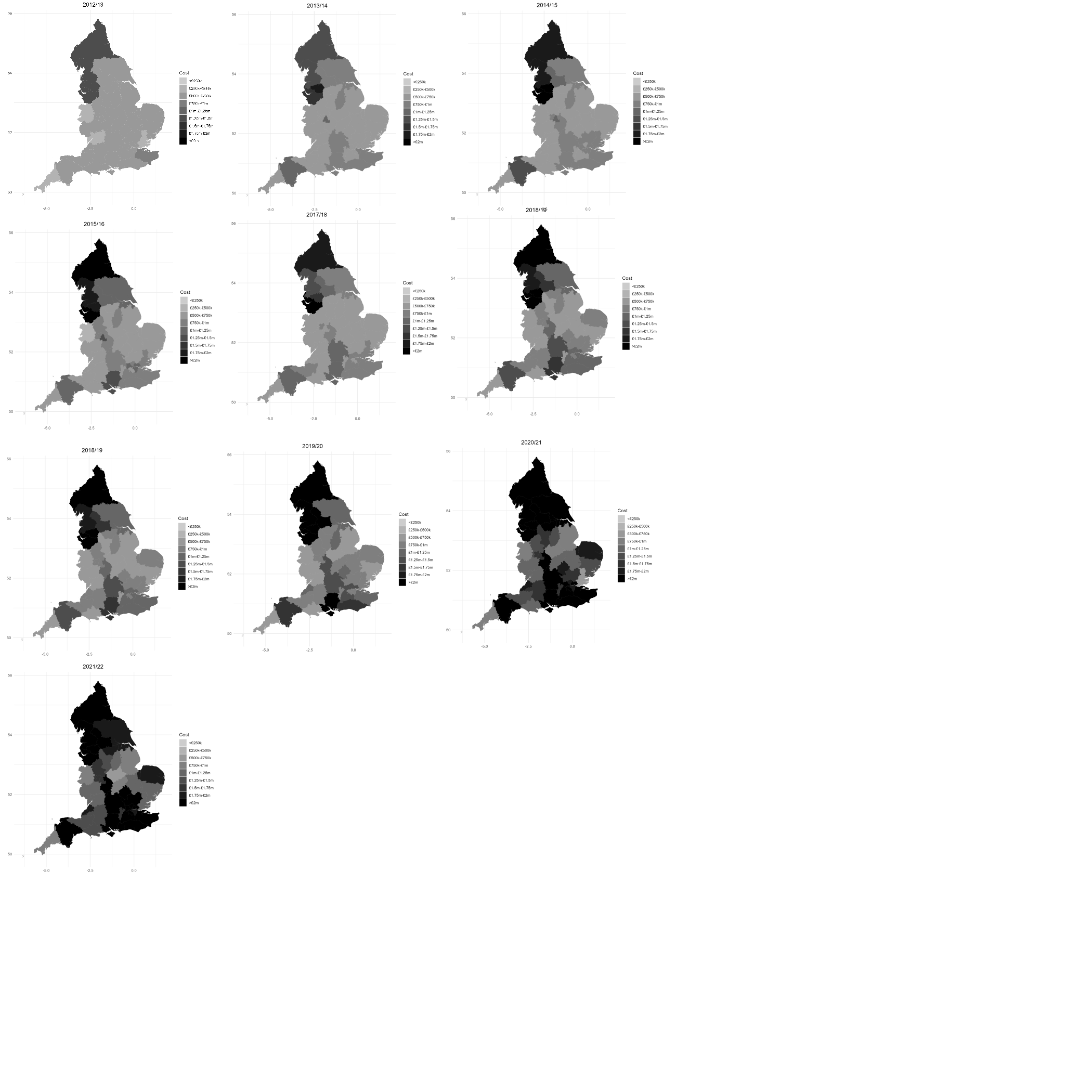

Supplement: online supplemental file 7 [file bmjopen-16-5-s007.png]
