# Supplementary material for: Cost of emergency hospital admissions to acute general wards for mental health problems among children and young people in England, 2012–2022: a retrospective observational study
Source: BMJ Open. 2026 May 19;16(5):e107143. doi: 10.1136/bmjopen-2025-107143 (PMC13202060; doi:10.1136/bmjopen-2025-107143)
Supplement: online supplemental file 8 [file bmjopen-16-5-s008.docx]

# Appendix

## Supplemental tables

Table A1: Share and number of admissions with missing unit cost before and after substitution (mental health admissions only)

Admissions with missing unit costs Before substitution After substitution

| Year | Total admissions | Numbers | Share (%) |  | Numbers | Share (%) |
| --- | --- | --- | --- | --- | --- | --- |
| 12/13 | 24197 | 99 | 0.41 |  | 82 | 0.34 |
| 13/14 | 30651 | 185 | 0.6 |  | 0 | 0 |
| 14/15 | 32407 | 8649 | 26.69 |  | 4 | 0.01 |
| 15/16 | 34891 | 9970 | 28.57 |  | 0 | 0 |
| 16/17 | 33868 | 10340 | 30.53 |  | 1 | 0 |
| 17/18 | 35647 | 862 | 2.42 |  | 13 | 0.04 |
| 18/19 | 37847 | 934 | 2.47 |  | 17 | 0.04 |
| 19/20 | 38236 | 909 | 2.38 |  | 60 | 0.16 |
| 20/21 | 35692 | 732 | 2.05 |  | 78 | 0.22 |
| 21/22 | 39925 | 346 | 0.87 |  | 103 | 0.26 |

Note: Some outdated HRG codes such as HRG4 for certain services were used in the HES data in some years. We have converted HRG4+ codes in NCC data to HRG4 where necessary to be compatible with HES data.

## Supplemental figures

Figure A1: Number of admissions of all-cause emergency paediatrics and emergency MH among CYP

[Figure A1 here]

(a) All cause (b) MH

Figure A2: Cost of all-cause emergency paediatrics and emergency MH among CYP (all costs are expressed in constant 2015 prices.)

[Figure A2 here]

(a) All cause (b) MH

Figure A3: Number of emergency mental health admissions of CYP by patient characteristics

[Figure A3 here]

Figure A4: Cost of admissions of emergency MH among CYP by patient characteristics (all costs are expressed in constant 2015 prices.)

[Figure A4 here]

Figure A5: Number of emergency MH among CYP by IMD quintile of the residence of the patient

[Figure A5 here]

Figure A6: Share of long stay admissions of total MH emergency admissions by IMD quintiles

[Figure A6 here]

Figure A7: Cost of admissions of emergency MH among CYP by ICB of the residence of the patient (all costs are expressed in constant 2015 prices.)

[Figure A7 here]
