# Supplementary material for: Cost of emergency hospital admissions to acute general wards for mental health problems among children and young people in England, 2012–2022: a retrospective observational study
Source: BMJ Open. 2026 May 19;16(5):e107143. doi: 10.1136/bmjopen-2025-107143 (PMC13202060; doi:10.1136/bmjopen-2025-107143)
Supplement: Supplementary file 9 [file bmjopen-2025-107143.reviewer_comments.pdf]

PEER REVIEW HISTORY

BMJ Open publishes all reviews undertaken for accepted manuscripts. Reviewers are asked to complete a checklist review form and are provided with free text boxes to elaborate on their assessment. These free text comments are reproduced below.

ARTICLE DETAILS

Title (Provisional)

The cost of emergency hospital admissions to acute general wards for mental health problems among children and young people in England, 2012–2022: a retrospective observational study.

Authors

Pilvar, Hanifa; Cornaglia, Francesca; Ward, Joseph L; Vazquez-Vazquez, Adriana; Phillips, Kirsty; Settle, Kate; Gibson, Faith; Nicholls, Dasha; Roland, Damian; Roberts, Helen; Viner, Russell M; Hudson, Lee

VERSION 1 - REVIEW

**Reviewer**	**1**
**Name**	**Awara, Mahmoud**
**Affiliation**	**Dalhousie University, Psychaitry**
**Date**	**03-Aug-2025**
**COI**	**None**

Thank you for inviting me to review this interesting and well-written study.

1. Page 7, Table 1, IMD quintile, in terms of how the four most deprived can be differentiated, did the author mean the next less deprived, middle, next… Could the author clarify the factors used in determining deprivation, e.g. Income, health, education, employment, etc..

2. On page 9, could the author write that the NHS is the National Health Service, as readers outside the UK would not know that, and also explain the abbreviation of the ICB?

3. Page 10, line 36 “The cost of paediatric emergency mental health admissions was 22.5 million Pounds in 2021/22 which rose to 87.3 million Pounds in constant 2015 prices (see Supplemental Figure A2)”. There is some ambiguity about this sentence; can the author rephrase it?

4. On page 13, “For instance, in 2012/13, admissions among CYP from the most deprived areas were 80% higher than those from the least deprived neighbourhoods. By 2021/22, this

gap had reduced to just 5%, indicating a rapid rise in admissions from the least deprived areas". Could the author explain why the gap has narrowed?

5. On page 15, the authors feel that the acute services are absorbing the burden of CYP admission despite being poorly equipped to do so; could the authors add evidence to this conclusion?

6. On page 15, line 8, the authors suggested that an alternative interventional community pathway, e.g. Crisis intervention, community care-based services, could be an alternative to acute and costly admissions. Are the authors aware of studies that looked at whether assertive outreach or crisis intervention has reduced the admission and length of stay of the CYP, to draw such a conclusion? If so, can this be added to strengthen the discussion?

7. On page 29, Figure 1, is the drop in the number of admissions and cost in 2020-2021, was that COVID-19 related?

8. In figure 3, would the authors be able to explain why the most deprived residence-related had the lowest cost in 2021-2022.

9. In Figure A2-page 36, despite the drop in the number of admissions in 2021-2022 in Figure A1-Page 34, the cost didn't proportionally drop but plateaued. Would the author explain that?

10. Age group 11-15, page 38, seems to have a high admission rate according to Figure A3. Is there any explanation?

11. Page 39, are female more prone to admissions due to high prevalence of self harm and borderline personality disorders compared to males? Could the author clarify reasons.

12. On page 40, white ethnicity showed the highest admission rate. Is this consistent with their representation in the sample studied? Is there an explanation as to why this was the case, e.g. were they more prone to self-harm or BPD?

13. Page 41, eating disorders were low in number of admissions but costly, is this related to the nature of the intervention, e.g. ICU admission, IV treatment, etc?

14. Can the figures be reduced and populated when feasible to allow visual comparison?

15. Page 47, figure A6, it shows drops in long stay admissions of all quintiles in the year 2019-202 except quintile 1 that dropped in 2021-2021 when others rose. Any explanation to that trend.

Thank you,

Reviewer **2**

Name **Usami, Masahide**

Affiliation **Kohnodai Hospital, National Center for Global Health and Medicine, Department of Child and Adolescent Psychiatrty**

**Date**	**04-Jan-2026**
**COI**	**None**

I have reviewed the manuscript entitled "Costs of admission of children and young people to acute medical wards for mental health problems in England from 2012 to 2022".
Below, we offer comments to ensure international accessibility, paying particular attention to the health system context, cost estimation methodology, and interpretation from a child and adolescent psychiatry perspective.
overview
This study uses linked Hospital Episode Statistics (HES) and national cost collection data to provide a valuable national analysis of trends and costs associated with emergency admissions for child and young people's mental health problems (CYP) in England. The findings—that costs rise sharply, particularly with eating disorders, self-harm, and prolonged stays, and that substantial socioeconomic and regional inequalities persist—are highly relevant for policy and service planning. However, for publication in an international journal, several aspects need to be strengthened, particularly with regard to terminology, description of the UK healthcare and payment system, international contextualization, scope and limitations of cost estimates, and interpretation of regional variation. Overall, I recommend significant revision.
Key Points
1. Clarification of terminology and research focus
This manuscript analyzes the costs associated with emergency admission of CYP with mental health problems to acute (general) wards, rather than admission to specialist psychiatric wards – a distinction that is important but may not be immediately clear to an international readership.
We recommend that you clearly emphasize in the title, abstract, and introduction that the setting is an acute care unit that serves as a de facto safety net for mental health crises.
The operational definition of hospitalization for self-harm (a primary diagnosis of injury/external cause with self-harm recorded as the first secondary diagnosis) varies between countries, limiting international comparability, and therefore needs to be clearly stated as an administrative definition.
2. Description of the UK healthcare and payment system
While Healthcare Resource Groups (HRGs), currencies, and national cost collections are explained, it should be clearly stated that the NCC figures represent average provider-unit costs, not reimbursement tariffs or actual payments.
For an international audience, please note that these costs reflect the perspective of healthcare providers within the tax-funded NHS, rather than direct patient charges, and therefore do not represent claims or patient-level expenditure. - To contextualize the geographic variation analysis, a brief explanation of the role of Integrated Care Boards (ICBs) - regional NHS bodies responsible for planning and allocating healthcare resources - is necessary.

3. Limited international comparisons
The global importance of child and adolescent mental health has been emphasized, but explicit international comparisons are limited.
The discussion would benefit from more clearly framing how these findings relate to other health systems.
For example: the difference between psychiatric bed availability and community crisis services
To what extent are acute care wards used for mental health crises in other countries?
How different insurance or reimbursement systems shape admission criteria and length of stay
Even if direct comparisons are not possible, making these contrasts explicit significantly strengthens international relevance.
4. Scope and Limitations of Cost Estimates
The manuscript should more prominently emphasize that estimated costs are based on admission numbers, HRG groups, and length of stay, and do not capture the following:
fluctuations in treatment intensity or staffing within the ward;
Costs outside of the inpatient episode (e.g., follow-up care, community services),
Whether it be the strain on families, disruption to education, or long-term economic impacts.
Costs to broader society - Splitting length of stay into less than two days and more than two days, while understandable for consistency with NCC data, hides substantial heterogeneity within long-stay hospitalizations (e.g., days vs. weeks).
This limitation should be more clearly highlighted in policy interpretations.
5. Regional differences and distribution of the professional workforce
This analysis shows clear geographic variation in costs among ICBs, but does not directly examine supply-side factors such as the availability of child and adolescent psychiatrists, specialist beds, CAMHS capacity, or crisis services.
As a result, causal interpretations remain speculative. We encourage strengthening the discussion and explicitly framing these findings as hypotheses about resource allocation and specialist workforce shortages, rather than implicit explanations. - Linking cost patterns to service capacity indicators, where feasible in future work, would significantly enhance policy impact.
Minor points
1. The implications of using the old HRG4 code and converting HRG4+ data should be briefly repeated in the main limitations section as well as in the supplementary material.
2. Differences between the number and costs of hospitalizations after COVID-19 caused by changes in diagnostic mix should be summarized more clearly in the executive summary or conclusions.
3. Figure captions should consistently specify that all costs are expressed in constant 2015 prices.

in conclusion
This is a high-quality, policy-relevant study that makes an important contribution to understanding the economic burden of the childhood mental health crisis in the UK. Addressing the points above, particularly those relating to the health system context, international frameworks and interpretation of regional variation, would significantly strengthen the manuscript for an international audience.

VERSION 1 - AUTHOR RESPONSE

Reviewer: 1- Dr. Mahmoud Awara, Dalhousie University

Comment 1. Page 7, Table 1, IMD quintile, in terms of how the four most deprived can be differentiated, did the author mean the next less deprived, middle, next… Could the author clarify the factors used in determining deprivation, e.g. Income, health, education, employment, etc.

Response 1: Thank you for this comment. We have revised the labelling of IMD categories in Table 1 to use standard quintile terminology (Quintiles 1–5, from least to most deprived) to improve clarity and avoid ambiguity between intermediate categories. We have also added a table footnote explaining how the Index of Multiple Deprivation is calculated in England, including the domains used and how quintiles were assigned (page 12 line 3). The footnote is: "Deprivation was measured using the Index of Multiple Deprivation (IMD), an area-level composite measure of relative deprivation in England. IMD is based on seven weighted domains: income, employment, education, health, crime, barriers to housing and services, and living environment. Patients were assigned to national IMD quintiles based on their area of residence, with Quintile 1 representing the least deprived 20% of areas and Quintile 5 the most deprived 20%."

Comment 2. On page 9, could the author write that the NHS is the National Health Service, as readers outside the UK would not know that, and also explain the abbreviation of the ICB?

Response2: Thank you for this comment. We have revised the manuscript to define NHS as the National Health Service (page 4 line 24) and to explain the abbreviation ICB as Integrated Care Board on page 9 line 1 to improve clarity for readers outside the UK.

Comment 3. Page 10, line 36 "The cost of paediatric emergency mental health admissions was 22.5 million Pounds in 2021/22 which rose to 87.3 million Pounds in constant 2015 prices (see Supplemental Figure A2)". There is some ambiguity about this sentence; can the author rephrase it?

Response3: There was a typo in this sentence. Thanks for mentioning it. We changed it to (page 12 line 21) "The cost of paediatric emergency mental health admissions was 22.5 million Pounds in 2012/13 which rose to 87.3 million Pounds in 2021/22. All costs are adjusted for the inflation using constant 2015 prices (see Supplemental Figure A2)."

Comment 4. On page 13, "For instance, in 2012/13, admissions among CYP from the most deprived areas were 80% higher than those from the least deprived neighbourhoods. By 2021/22, this gap had reduced to just 5%, indicating a rapid rise in admissions from the least deprived areas". Could the author explain why the gap has narrowed?

Response4: To clarify, we have added the following sentence (page 16 line 5): "This narrowing gap reflects a more rapid increase in admissions among children and young people from the least deprived areas over time, rather than a decline in admissions from the most deprived neighbourhoods. This trend is descriptive and may be driven by multiple factors, including changes in help-seeking behaviour, thresholds for admission, and availability of community-based mental health services across socioeconomic groups."

Comment 5. On page 15, the authors feel that the acute services are absorbing the burden of CYP admission despite being poorly equipped to do so; could the authors add evidence to this conclusion?

Response5: Thank you for this comment. We have strengthened this statement by explicitly citing evidence on rising demand for community and specialist child and adolescent mental health services, increasing waiting times, and unmet need within CAMHS (page 18 line 6). Here is the added paragraph: "Evidence indicates that acute hospital services are increasingly managing children and young people presenting in mental health crisis, despite being primarily designed for physical health care rather than specialist mental health treatment. In England, referrals to child and adolescent mental health services (CAMHS) more than doubled between 2017 and 2022, signalling a substantial increase in underlying need. While investment in children and young people's mental health services has risen over this period, it has not matched the growth in demand. Consequently, a growing share of referrals have been closed before treatment, reversing previous improvements, and waiting times for those accepted into care have increased. These pressures on community and specialist services may be contributing to more children and young people presenting in crisis to emergency departments and being admitted to acute medical wards, which may partly account for the rising admissions and costs observed in this study."

Comment 6. On page 15, line 8, the authors suggested that an alternative interventional community pathway, e.g. Crisis intervention, community care-based services, could be an alternative to acute and costly admissions. Are the authors aware of studies that looked at whether assertive outreach or crisis intervention has reduced the admission and length of stay of the CYP, to draw such a conclusion? If so, can

this be added to strengthen the discussion?

Response 6: Thank you for this comment. We have revised the Discussion to strengthen the evidential basis for our statement regarding community-based crisis pathways. We now explicitly cite evidence from a systematic review examining alternatives to inpatient admission for children and young people experiencing mental health crises and clarify the limitations of the current evidence base (page 18 line 17). The revised paragraph now reads: "Associated high costs are an important part of the argument for strengthening and redeveloping pathways of care as a priority. This could include greater investment in community-based services, early intervention programmes, and crisis care alternatives. Evidence on alternatives to admission to acute hospital settings for children and young people experiencing a mental health crisis remains limited and heterogeneous. However, a systematic review by Clisu et al. identified some evidence that in-home interventions, particularly multisystemic therapy, may be associated with improved psychological outcomes and shorter lengths of hospital stay, although many children and young people still required admission. Overall, the authors concluded that no single intervention could be recommended as a substitute for inpatient care, highlighting the need for closer alignment between community and inpatient services and for further high-quality research evaluating community-based crisis interventions."

Comment 7. On page 29, Figure 1, is the drop in the number of admissions and cost in 2020-2021, was that COVID-19 related?

Response 7: Yes, the drop in the number of admissions and the change in costs during 2020–2021 are likely related to the COVID-19 pandemic. This is already noted in the manuscript. On page 13 line 2: "There was a noticeable spike in mental health admission costs after 2019/20, followed by a flattening after 2020/21, coinciding with the COVID-19 pandemic."

Comment 8. In figure 3, would the authors be able to explain why the most deprived residence-related had the lowest cost in 2021-2022.

Response 8: Yes, this pattern is explained in the Results section on page 15 line 22 onwards. We note that while children and young people from the most deprived areas accounted for the highest costs before the COVID-19 pandemic, this pattern changed in 2021/22. One contributing factor is a decline in the share of long-stay admissions, which are the most costly, among children and young people from the most deprived areas after the pandemic, alongside an increase in the share of long-stay admissions among those from the least deprived neighbourhoods.

Comment 9. In Figure A2-page 36, despite the drop in the number of admissions in 2021-2022 in Figure A1-Page 34, the cost didn't proportionally drop but plateaued. Would the author explain that?

Response 9: Thank you for this comment. The plateau in costs despite a reduction in the number of admissions reflects a change in the composition of admissions. Specifically, there was a relative decline in short-stay admissions and a concurrent increase in long-stay admissions, which are substantially more costly. We added this paragraph to page 13 line 22: "The plateau in costs observed after 2020–2021, despite a reduction in total admissions in Figure 1, reflects a change in the composition of admissions. Specifically, following the COVID-19 pandemic there was a decline in short-stay admissions and a concurrent increase in long-stay admissions, which are substantially more costly. This shift in case mix explains the observed divergence between trends in total admissions and overall costs shown in Figure 1."

Comment 10. Age group 11-15, page 38, seems to have a high admission rate according to Figure A3. Is there any explanation?

Response 10: Thank you for this comment. Our study is descriptive in nature and is not designed to identify causal relationships between age and mental health admissions. While Figure A3 shows higher admission rates among children and young people aged 11–15, we cannot determine the underlying reasons for this pattern using administrative data. This likely reflects a combination of developmental, clinical, and service-related factors, which are beyond the scope of the current analysis.

Comment 11. Page 39, are female more prone to admissions due to high prevalence of self harm and borderline personality disorders compared to males? Could the author clarify reasons.

Response 11: Thank you for this comment. Our study is descriptive and cannot determine causal reasons for sex differences in admission rates. However, to clarify potential contributing factors, we have added a paragraph to the Discussion on page 17 line 17: "This suggests that conditions requiring intensive and prolonged care are major cost drivers within CYP mental health emergency admissions, particularly among females, where eating disorders are much more common." We have added the following reference to support this statement (line 16 page 25): Striegel-Moore RH, Rosselli F, Perrin N, DeBar L, Wilson GT, May A, Kraemer HC. Gender differences in the prevalence of eating disorder symptoms. International Journal of Eating Disorders. 2009;42(5):471–474.

Comment 12. On page 40, white ethnicity showed the highest admission rate. Is this consistent with their representation in the sample studied? Is there an explanation as to why this was the case, e.g. were they more prone to self-harm or BPD?

Response 12: Thanks for pointing this out. We have explained this on page 15 line 2 as follows: "However, CYP with white background have higher admission rate than their share in the study group which might be due to better service access for this group."

Comment: 13. Page 41, eating disorders were low in number of admissions but costly, is this related to the nature of the intervention, e.g. ICU admission, IV treatment, etc?

Response 13: Our study is not able to examine the specific clinical interventions delivered during hospitalisations, such as intensive care use or intravenous treatments, as this information is not available in the administrative data used. As such, we cannot determine whether particular treatments explain the higher costs associated with eating disorder admissions. However, as noted in the Results section (page 17, line 17), admissions for eating disorders are typically associated with substantially longer lengths of stay, which is a key driver of higher costs in our analysis. The higher costs therefore reflect prolonged hospitalisation rather than specific treatment modalities, which is beyond the scope of the current study.

Comment 14. Can the figures be reduced and populated when feasible to allow visual comparison?

Response 14: Thank you for this suggestion. We have carefully considered whether the figures could be further reduced or combined. However, given the number of dimensions explored (time, diagnosis, length of stay, deprivation, and geography), further consolidation would substantially reduce clarity and limit the ability to interpret key patterns. We therefore felt it was important to retain the current figure structure to ensure transparency and interpretability of the results. When figures are arranged into panels during the publication process, we expect that visual comparison across related results will be clearer and easier to follow.

Comment 15. Page 47, figure A6, it shows drops in long stay admissions of all quintiles in the year 2019-202 except quintile 1 that dropped in 2021-2021 when others rose. Any explanation to that trend.

Response 15: Thank you for this comment. We are not able to provide explanations for year-by-year fluctuations in the share of long-stay admissions across deprivation quintiles. While Figure A6 shows some short-term variation between individual years, the key pattern we observe is a general decline in the share of long-stay admissions among children and young people from the most deprived areas, alongside an upward trend in the least deprived areas. This pattern is described in the Results section on page 16 line 11.

Reviewer: 2- Dr. Masahide Usami, Kohnodai Hospital, National Center for Global Health and Medicine

Comment 1. Clarification of terminology and research focus
This manuscript analyzes the costs associated with emergency admission of CYP with mental health problems to acute (general) wards, rather than admission to specialist psychiatric wards – a distinction that is important but may not be immediately clear to an international readership.
We recommend that you clearly emphasize in the title, abstract, and introduction that the setting is an acute care unit that serves as a de facto safety net for mental health crises. The operational definition of hospitalization for self-harm (a primary diagnosis of injury/external cause with self-harm recorded as the first secondary diagnosis) varies between countries, limiting international comparability, and therefore needs to be clearly stated as an administrative definition.

Response 1: Thank you so much for your comment. We agree that it is important to be clear about the terminology used. We have changed the title to "The cost of emergency hospital admissions to acute general wards for mental health problems among children and young people in England, 2012–2022: a retrospective observational study". We also have cleared in the abstract in the setting and participant section that we are talking about acute wards. We acknowledged that our definition of self-harm is administrative and added this part to page 7 line 18 "This operational definition reflects English administrative coding practices and may limit direct comparability with studies from other countries that use different coding conventions."

Comment 2. Description of the UK healthcare and payment system
While Healthcare Resource Groups (HRGs), currencies, and national cost collections are explained, it should be clearly stated that the NCC figures represent average provider-unit costs, not reimbursement tariffs or actual payments.
For an international audience, please note that these costs reflect the perspective of healthcare providers within the tax-funded NHS, rather than direct patient charges, and therefore do not represent claims or patient-level expenditure. - To contextualize the geographic variation analysis, a brief explanation of the role of Integrated Care Boards (ICBs) - regional NHS bodies responsible for planning and allocating healthcare resources - is necessary.

Response 2: Thank you so much for your comment. We have clarified on page 6 line 7 that the cost is not patient level cost and added the following paragraph in description of NCC data: "We used the National Cost Collection (NCC) database to estimate the cost of admissions due to mental health concerns identified within HES. These data provide national average unit costs for NHS services derived from cost data submitted by NHS trusts. These figures reflect the average cost of delivering care from the perspective of healthcare providers and do not represent reimbursement tariffs, actual payments, insurance claims, or patient-level charges. As the National Health Service is a tax-funded system, patients do not incur direct charges for care, and costs therefore represent resource use within the healthcare system rather than

individual expenditure."

We also have added a description of ICBs to the geographic variation section as follows (page 9 line 2): "ICBs are statutory NHS organisations responsibley for planning and arranging NHS services within defined geographic areas. ICBs bring together NHS trusts, general practices, local authorities, and other partners to set strategic priorities, plan health services, allocate resources and oversee performance for the populations they serve."

Comment 3. Limited international comparisons
The global importance of child and adolescent mental health has been emphasized, but explicit international comparisons are limited.
The discussion would benefit from more clearly framing how these findings relate to other health systems. For example: the difference between psychiatric bed availability and community crisis services. To what extent are acute care wards used for mental health crises in other countries? How different insurance or reimbursement systems shape admission criteria and length of stay even if direct comparisons are not possible, making these contrasts explicit significantly strengthens international relevance.

Response 3: Thank you for this thoughtful comment. We have revised the Discussion to more explicitly situate our findings within an international context and to clarify how differences in health system organisation may shape the use of acute care for child and adolescent mental health crises. We have added the following paragraph to the discussion section (page 19 line 17): "Although our findings are based on data from England, which has a tax-funded National Health Service with no direct patient charges, they are likely to have relevance beyond the UK. Direct international comparisons are challenging because health systems differ substantially in the availability of specialist inpatient mental health units, the provision of community-based crisis services, and the role of insurance or reimbursement mechanisms in shaping admission thresholds and length of stay. Published data on admissions of children and young people to acute medical wards for mental health crises in other countries are limited, and there is a paucity of longitudinal analyses outside the UK. However, a recent systematic review identified evidence of increasing mental health-related admissions among children and young people across several geographical settings, suggesting that rising demand for acute care is not unique to England. Given the global increase in the incidence of child and adolescent mental health difficulties, it is plausible that acute care services in other high-income health systems are experiencing similar pressures, despite differences in service configuration. This underscores the importance of replicating similar analyses in other countries to better understand how health system design influences admission patterns, length of stay, and costs."

Comment 4. Scope and Limitations of Cost Estimates The manuscript should more prominently emphasize that estimated costs are based on admission numbers, HRG groups, and length of stay, and do not capture the following: fluctuations in treatment intensity or staffing within the ward; Costs outside of the inpatient episode (e.g.,

follow-up care, community services), Whether it be the strain on families, disruption to education, or long-term economic impacts. Costs to broader society - Splitting length of stay into less than two days and more than two days, while understandable for consistency with NCC data, hides substantial heterogeneity within long-stay hospitalizations (e.g., days vs. weeks). This limitation should be more clearly highlighted in policy interpretations.

Response 4: Thank you for this comment. We have revised the Discussion and Study limitations sections to more prominently emphasise the scope and limits of our cost estimates. The limitation section reads (page 21 line 19): "Furthermore, our cost estimates rely on national average unit costs, which may obscure local variations in spending and service efficiency [17]. Future research could explore trust-level variations in spending to better understand efficiency differences across the NHS. Additionally, our study primarily focuses on direct hospital costs and does not account for long-term economic consequences, such as increased primary care utilization following emergency admissions. Given the evidence that adolescent mental health issues have long-term financial impacts [11], future research should explore the broader economic costs of CYP mental health crises, including their effects on education, employment, and social care systems. Length of stay was categorised as less than two days or two days or more to align with National Cost Collection reporting. While this distinction captures broad differences in resource use, it conceals substantial heterogeneity within long-stay admissions, which may range from a few days to several weeks. As a result, policy interpretations of cost differences should recognise that long-stay admissions represent a diverse group of hospitalisations with potentially very different clinical pathways and resource requirements."

Comment 5. Regional differences and distribution of the professional workforce
This analysis shows clear geographic variation in costs among ICBs, but does not directly examine supply-side factors such as the availability of child and adolescent psychiatrists, specialist beds, CAMHS capacity, or crisis services.
As a result, causal interpretations remain speculative. We encourage strengthening the discussion and explicitly framing these findings as hypotheses about resource allocation and specialist workforce shortages, rather than implicit explanations. - Linking cost patterns to service capacity indicators, where feasible in future work, would significantly enhance policy impact.

Response 5: Thank you for this comment. We agree that our analysis does not directly examine supply-side factors such as specialist workforce availability, psychiatric bed capacity, or community mental health service provision. We have revised the Discussion added the following paragraph (page 20 line 9): "The observed geographic variation in costs across ICBs should be interpreted as descriptive rather than causal. Our analysis does not directly measure supply-side factors such as the availability of child and adolescent psychiatrists, specialist mental health beds, community CAMHS capacity, or crisis response services. As such, differences in costs between areas should be viewed as hypotheses regarding variation in service configuration, resource allocation, and specialist workforce capacity, rather than as evidence of specific mechanisms. Future research linking hospital admission costs to

local indicators of mental health service capacity, workforce availability, and community crisis provision would substantially strengthen understanding of how system-level factors shape admission patterns, length of stay, and costs, and would enhance the policy relevance of these findings."

Minor points

Comment 1. The implications of using the old HRG4 code and converting HRG4+ data should be briefly repeated in the main limitations section as well as in the supplementary material.

Response: We have stated the HRG4 to HRG4+ conversion as a limitation in the main text (page 21 line 15). We have also added a footnote to Table A1 in the supplementary material section (page 27 line 1).

Comment 2. Differences between the number and costs of hospitalizations after COVID-19 caused by changes in diagnostic mix should be summarized more clearly in the executive summary or conclusions.

Response 2: We have added the following statement to the result section in abstract (page 2 line 4): "Following the COVID-19 pandemic, total admission numbers declined, but overall costs remained high due to a shift in diagnostic mix towards conditions associated with longer hospital stays and higher per-admission costs."

Comment 3. Figure captions should consistently specify that all costs are expressed in constant 2015 prices.

Response 3: We have added the requested expression to the caption of all cost figures.

VERSION 2 - REVIEW

Reviewer 2

Name Usami, Masahide

Affiliation Kohnodai Hospital, National Center for Global Health and Medicine, Department of Child and Adolescent Psychiatrty

Date 17-Feb-2026

COI

Thank you for your hard work on these revisions.

I appreciate the thoroughness with which you addressed the points I raised. In particular, the new section on the limitations of cost estimates and the nuanced interpretation of geographic variation provide a very balanced and insightful perspective that will be highly valuable to policymakers.

The manuscript has been significantly enhanced through this process, and I believe it makes a substantial contribution to the field of child and adolescent mental health. I am fully satisfied with the current version.

I look forward to seeing this published in BMJ Open.
